# Development and Validation of an Immune-Related Prognostic Signature for Ovarian Cancer Based on Weighted Gene Coexpression Network Analysis

**DOI:** 10.1155/2020/7594098

**Published:** 2020-12-10

**Authors:** Yuanyuan An, Qing Yang

**Affiliations:** Gynecology Department in Shengjing Hospital of China Medical University, Shenyang, Liaoning Province, China

## Abstract

**Background:**

Ovarian cancer is one of the most lethal diseases of women. The prognosis of ovarian cancer patients was closely correlated with immune cell expression and immune responses. Therefore, it is important to identify a robust prognostic signature, which correlates not only with prognoses but also with immune responses in ovarian cancer, thus, providing immune-related patient therapies.

**Methods:**

The weighted gene coexpression network analysis (WGCNA) was used to identify candidate genes correlated with ovarian cancer prognoses. Univariate and multivariate Cox regression analyses were used to construct the prognostic signature. The Kaplan-Meier method was used to predict survival, and the immune-related bioinformatics analysis was performed using the R software. The relationship between the signature and clinical parameters was analyzed with the GraphPad Prism 7 and SPSS software.

**Results:**

Gene expression from The Cancer Genome Atlas dataset was used to perform the WGCNA analysis, and candidate prognostic-related genes in patients with ovarian cancer were identified. According to the Cox regression analysis, the prognostic signature was constructed, which divided patients into two groups. The high-risk group showed the least favorable prognosis. Three independent cohorts from the Gene Expression Omnibus (GEO) database were used for the validation studies. According to the immune analyses, the GEO database signatures were significantly correlated with the immune statuses of ovarian cancer patients. By analyzing the combination of the prognostic signature and total mutational burden (TMB), ovarian cancer patients were divided into four groups. In these groups, memory B cell, resting memory CD4 T cell, M2 macrophage, resting mast cell, and neutrophil were found with significant distinctions among these groups.

**Conclusions:**

This novel signature predicted the prognosis of ovarian cancer patients precisely and independently and showed significant correlations with immune responses. Therefore, this prognostic signature could indicate targeted immunotherapies for ovarian cancer patients.

## 1. Background

Ovarian cancer is one of the most common and lethal gynecologic tumors. Early detection of ovarian cancer is difficult because ovaries are located deep within the pelvic cavity and are small, and there is a lack of obvious symptoms. Current studies have shown that the tumor microenvironment has a dominant role in cancer progression, especially in regard to the immune cell microenvironment [1]. Ovarian cancer genes have high mutational rates closely correlated with immune status disorders, which further promote tumor progression [2].

In tumor-related research, many scientists have looked for differentially expressed genes (DEGs) between tumor and normal tissues, but correlations among the genes have been ignored. The weighted gene coexpression network analysis (WGCNA) is an algorithm that uses R packages in R software to build a scale-free network for the exploration of weighted correlations among gene clusters and phenotype-related modules [3, 4]. Related genes and hub genes can be identified to find candidate biomarkers or therapeutic targets. WGCNA identifies distinct aspects of coexpression networks and various biologic processes, particularly in cancer patients [5].

Recently, studies showed that immune responses have close relationships with ovarian cancer progression [6]. At present, some preclinical trials targeting immune cells in the ovarian cancer microenvironment have been performed. Programmed cell death protein 1 (PD-1) is an important immunosuppressive protein expressed on T cells. Liu et al. reported that the combination of PD-1 immune checkpoint inhibitors, nivolumab and bevacizumab, showed greater activity in relapsed platinum-sensitive ovarian cancer patients than when using either inhibitor alone [7]. Signal regulatory protein *α* (SIRP*α*) works as an immunosuppressive receptor on macrophages and, when combined with the CD47 ligand on cancer cells, sends out a “don't eat me” signal, inhibiting the phagocytic activity of macrophages. Huang et al. reported that the oncolytic adenovirus carrying the SIRP*α*-IgG1 Fc fusion gene could block CD47 signaling in ovarian cancer cells, therefore, increasing macrophage infiltration and killing ovarian cancer cells [8]. In addition, the total mutational burden (TMB) was also correlated with cancer prognoses and affected immune responses in the tumor microenvironment [9].

In this study, we developed a novel prognostic signature based on WGCNA, which divided patients into two groups according to large-scale expression datasets. In this study, due to the close relationship between the prognostic signature and immune microenvironments in ovarian cancer patients, we investigated the potential roles that immune cells play in ovarian cancer microenvironments according to the identified signature.

## 2. Results

### 2.1. Construction of the Ovarian Cancer Coexpression Modules

**Figure 1:**
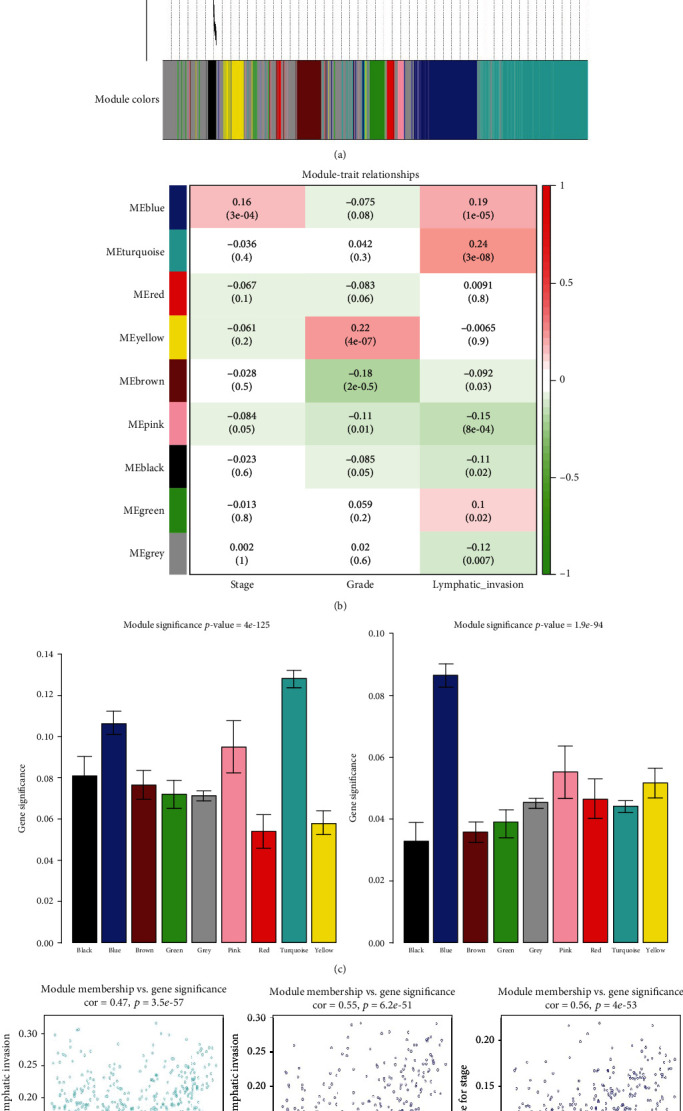
Coexpression module construction of ovarian cancer data arrays of TCGA dataset. (a) Clustering dendrograms of the top 25% variant genes based on a dissimilarity measure (1-TOM). (b) A heatmap of the correlations between module eigengenes and ovarian cancer clinical traits. The row represents distinct eigengene modules, and the column represents distinct clinical traits. The corresponding correlation and *p* value are shown in each cell. The table is color-coded by correlation according to the color legend. (c) Bar graphs showing the distribution of the average gene significance and error in the modules associated with ovarian cancer progression. The left graph shows the association between modules and ovarian cancer lymphatic invasion, and the right graph shows the association between modules and ovarian cancer stages. (d) Scatter plots of gene significance (GS) for lymphatic invasion or clinical stage vs. module membership (MM) in the turquoise or blue modules using linear regression.

The WGCNA package in R language was used to perform the clustering analyses of ovarian cancer samples from TCGA dataset shown in Figure S1(a). Of the 537 samples, 525 revealed no significant differences in the clustering analysis. The corresponding clinical parameters are shown in Figure S1(b). The power value, representing the most critical parameter, was screened out to form a scale-free network, which influenced the scale independence and mean connectivity of the coexpression module. In our study, we set *β* = 3 (scale − free = 0.94) as the power value with a scale independence of up to 0.9, and higher mean connectivity (Figure S2). Subsequently, *β* = 3 was used to construct the coexpression modules, of which nine different ovarian cancer modules were identified (including a grey module) (Table S1^1^). Genes with similar expression patterns were placed in one module using average linkage clustering, and the first 25% most variant genes were used from the 525 samples (Figure 1(a)).  Figure 1(b) shows the correlation between the coexpression modules and clinical traits. As is commonly understood, clinical stage, clinical grade, and the presence of lymphatic invasion correlate with ovarian cancer prognoses. For this study, we chose modules with gene numbers >200 and *p* values < 0.001 to study further. Therefore, we selected turquoise modules, which showed the most relevance with ovarian cancer lymphatic invasion and blue modules, which showed the most relevance with ovarian cancer stages (Figure 1(c)). In addition, gene significance plots vs. module memberships are shown in Figure 1(d). From the blue and turquoise modules, 1648 genes were further analyzed using Gene Ontology (GO) and Kyoto Encyclopedia of Genes and Genomes (KEGG) analyses, as shown in Tables S2-S5. Notably, the biologic pathway analysis showed that these genes significantly correlated with immune responses, indicating that immune responses participate in ovarian cancer progression and prognoses (Figure S3).

### 2.2. Identification of a Novel Ovarian Cancer Prognostic Signature

A univariate Cox regression analysis was used to investigate the prognostic role of the1648 candidate genes from turquoise and blue modules. The top 15 genes with a *p* value of < 0.001 were used for further analyses (Table 1). In addition to the univariate Cox regression analysis, the Kaplan-Meier method was used to predict overall survival (OS) of the 15 candidate genes. The results showed that all of the genes were significantly correlated with ovarian cancer prognoses and were consistent with the results of the univariate cox regression analyses (Figure S4). Next, the multivariate Cox regression analyses were used to construct prognostic signatures, and 4 genes were chosen using the following equation: Risk score = (0.11483 × *CH*25*H* expression) + (0.22472 × *HSPB*7  expression)–(0.28916 × *LOC*158830 expression) + (0.21726 × *PPM*2*C* expression).

**Figure 2:**
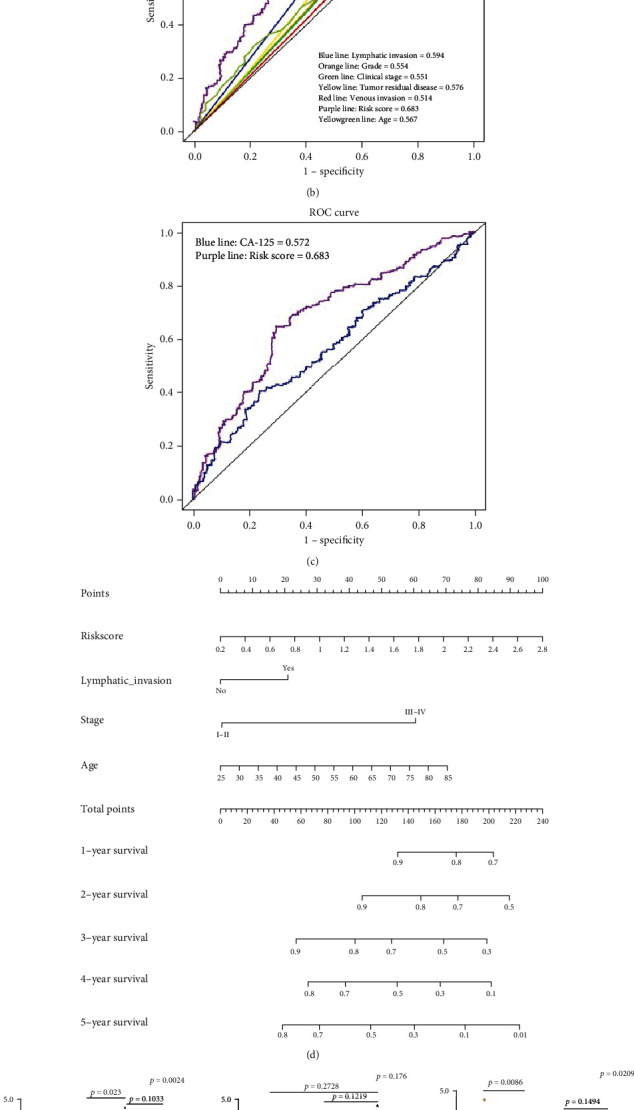
Identification of a prognostic signature in ovarian cancer patients. (a) The high-risk group showed unfavorable overall survival (OS) for ovarian cancer patients using the Kaplan-Meier method. (b) The receiver operating curve (ROC) 3-year OS analysis looking at the signature with other clinical factors using survival ROC in R package. (c) The ROC 3-year OS analysis looking at the signature with the CA-125 gene using survival ROC in R package. (d) A prognostic nomogram of ovarian cancer patients combined the signature and clinical parameters. (e) Correlations between the signature and clinical factors of ovarian cancer patients using one-way ANOVA.

According to the signature risk scores, patients were divided into two groups, namely high-risk and low-risk groups. By investigating the prognostic value of the signatures, the high-risk group was found to have shorter survival times than the low-risk group (Figure 2(a), *p* < 0.001). Stratified survival analyses showed that the prognostic signature significantly correlated with OS in ovarian cancer patients according to the clinical parameters (Figure S5). These analyses indicated that the signatures could precisely predict prognoses and did not need the clinical parameter information. Moreover, to investigate the accuracy of the identified signature, a 3-year receiver operating characteristic (ROC) curve analysis was performed. The ROC of the signature was 0.683, which was significantly higher than that of the prognostic-related clinical parameters (Figure 2(b)). Clinically, the CA-125 gene was found to be a highly sensitive biomarker for ovarian cancer diagnoses. However, the ROC of the CA-125 gene was 0.572, which was significantly lower than that of the signature (Figure 2(c)). A nomogram was constructed to predict the clinical survival of ovarian cancer patients by combining the signature with other clinical parameters (Figure 2(d)). The signature risk score was found to be closely correlated with the successful outcomes of primary therapies (Figure 2(e)). Through the examination of venous invasion, tumor residual disease, and clinical stages in ovarian cancer patients, the signature could precisely predict ovarian cancer prognoses.

In Table 2, the univariate and multivariate Cox regression analysis was used to test whether this signature could act as an independent prognostic factor for ovarian cancer. The results showed that it could act as an independent factor when adjusted for age, stage, grade, tumor residual disease, and lymphatic and venous invasion. To clarify the relationship between the signature and clinical parameters, the samples were divided into two groups. The signature was found to be significantly correlated with the clinical stage, residual tumor size, venous invasion, therapeutic outcome, and patient cancer status (Table 3).

To investigate the distinct biologic features between the high-risk and low-risk groups, DEGs with a fold change of >1.5 and a *p* value of < 0.05 were chosen (Table S6). Using the DAVID Gene Functional Classification software, we analyzed 170 candidate genes. The results indicated that the biologic process (BP) enrichments were significantly correlated with immune responses (Figure S6). The results showed that the identified prognostic signature might be closely related to ovarian cancer patient immune responses, thus, providing potential immunotherapy for these patients.

### 2.3. Validation of the Prognostic Signature with Independent Cohorts of Ovarian Cancer

**Figure 3:**
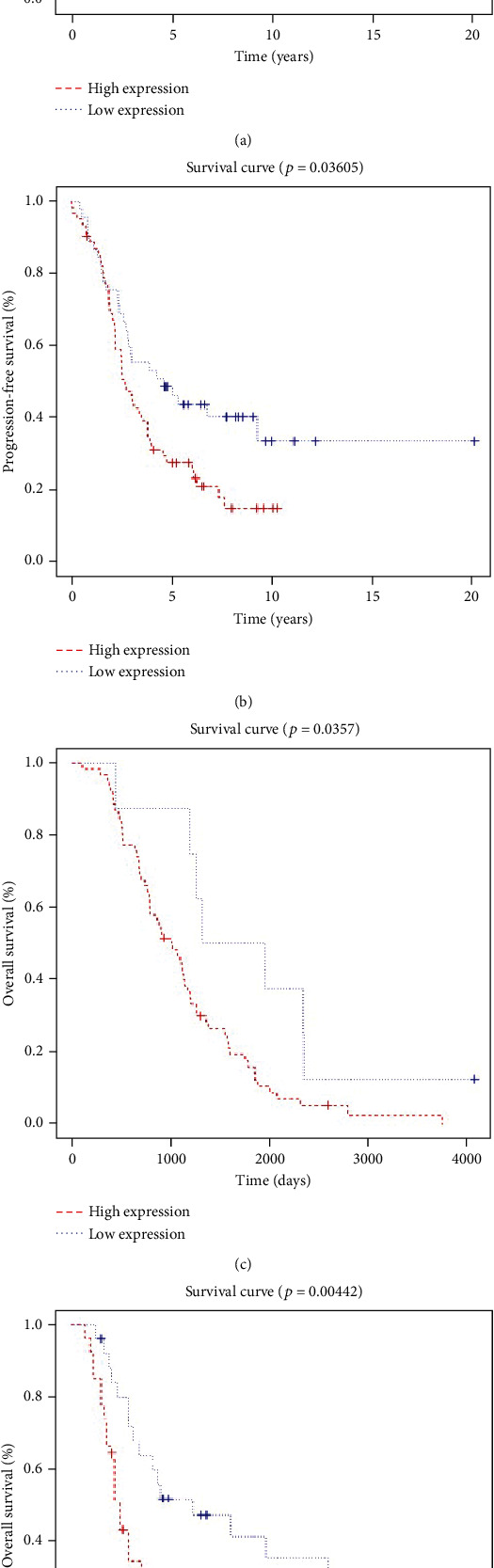
Validation of the prognostic signature with independent cohorts. (a) The high-risk group showed an unfavorable overall survival (OS) of ovarian cancer patients in the GSE26193 dataset using the Kaplan-Meier method. (b) In the GSE26193 dataset, the patients have been divided into two groups according to the risk scores. Compared with the low-risk group, the high-risk group showed an unfavorable progression-free survival of the ovarian cancer patients in the GSE26193 dataset using the Kaplan-Meier method. (c) In the GSE63885 dataset, the patients have been divided into two groups according to the risk scores. Compared with the low-risk groups, the high-risk group showed an unfavorable OS of the ovarian cancer patients in the GSE63885 dataset using the Kaplan-Meier method. (d) The high-risk group showed an unfavorable OS of ovarian cancer patients in the GSE18520 dataset using the Kaplan-Meier method.

To validate the signature of the ovarian cancer datasets derived from The Cancer Genome Atlas (TCGA) dataset, we downloaded GSE26193, GSE63885, and GSE18520 from the GEO database to represent three independent cohorts. According to the signature risk scores, the patients were divided into two groups. In these three datasets, patients with higher risk scores showed worse prognoses as expressed by shorter OS times than patients with lower risk scores (Figure 3). According to the Cox regression analysis of the GSE26193 dataset, this signature was an independent prognostic factor for ovarian cancer (Table S7).

In Table S8, we analyzed the relationship between the signature and the clinical parameters. However, in the GSE26193 and GSE63885 datasets, no clinical parameters were found that correlated with the signature, which could have been due to the limited number of samples in these two datasets.

### 2.4. The Prognostic Signature Correlates with Immune Cell Expression in the Ovarian Cancer Microenvironment

**Figure 4:**
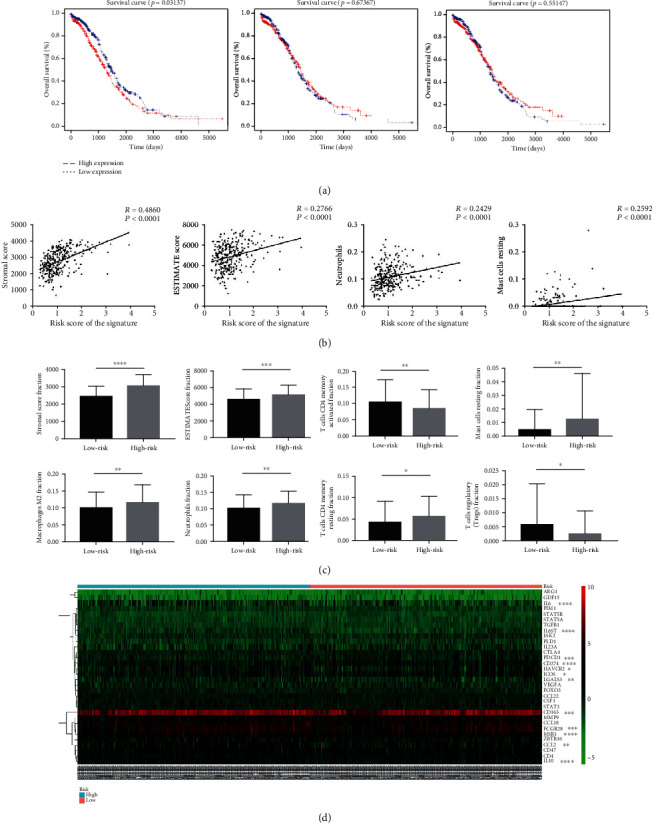
The correlation between the prognostic signature and immune cells of the tumor microenvironment in ovarian cancer data samples. (a) The Kaplan-Meier curves for overall survival of stromal cells, immune cells, and the estimation of stromal and immune cells in malignant tumor tissues using expression data (ESTIMATE) in ovarian cancer patients. (b) Pearson's correlation analyses between the signature and the immune cells. (c) Column plots show the correlation between the signature and the expression of immune cells. (d) A heatmap of the signature with immune-related genes using pheatmap in the R package. ∗*p* < 0.05; ∗∗*p* < 0.01; ∗∗∗*p* < 0.001; ∗∗∗∗*p* < 0.0001.

Tumor microenvironments play an important role in regulating ovarian cancer progression. According to the above analyses, we found that the prognostic signature might have a close relationship with the immune responses of ovarian cancer patients. In Figure 4(a), we showed that stromal cell expression in the tumor microenvironment correlated with ovarian cancer prognoses. The Pearson correlation analysis showed that the signature was positively correlated with stromal scores, estimation of stromal and immune cells in malignant tumor tissues using expression data (ESTIMATE) scores, and neutrophil and resting mast cell expression with an *R* > 0.2 (Figure 4(b)). According to the signature risk score, the samples were divided into two groups. The relationship between immune cells and the signature were tested, and the results are shown in the bar charts of Figure 4(c). These two methods showed similar results, which proved that the signature correlated significantly with stromal score expression, ESTIMATE scores, and neutrophils and resting mast cell expression. In addition, we selected a few immune checkpoint-related genes to further investigate the signature relationships (Figure 4(d)). The results showed some immune-related genes, such as LGALS3, PDCD1, IL6, IL6ST, CD163, FCGR2B, MSR1, HAVCR2, ICOS, IL10, and CCL2, were significantly correlated with the signature.

### 2.5. The Correlation between the Signature and the Immune Status of Ovarian Cancer Patients

The immune status of patients is well-known to play an important role in cancer progression. Yue et al. showed that some clinical parameters of ovarian cancer correlated with the expressions of some immune cells, such as grade of ovarian cancer with M1 macrophages and activated NK cells [10]. In addition, the expression of M0 and M1 macrophages also correlated with the overall survival of ovarian cancer patients [11]. Therefore, macrophages might play an important role in ovarian cancer progression.

**Figure 5:**
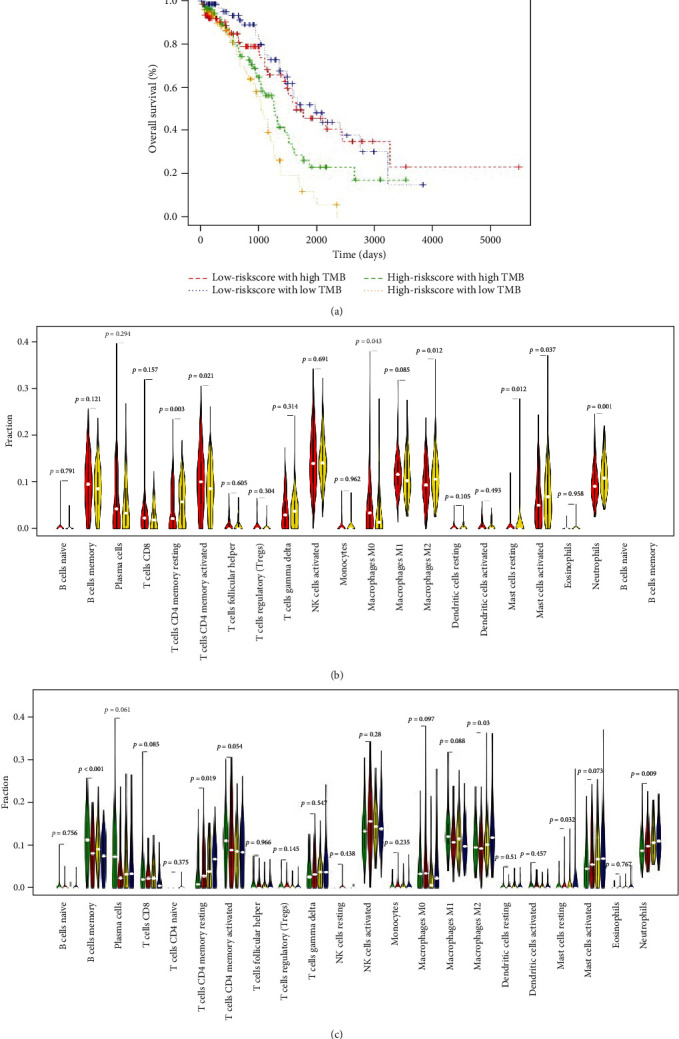
The correlation between signature and immune status in ovarian cancer patients. (a) The Kaplan-Meier curve showing OS after combining the signature with TMB expression. (b) The relationship between immune cells and the signature on a string map showed that the signature significantly correlated with the expression of T cells CD4 memory resting, T cells CD4 memory activated, M0 macrophages, M2 macrophages, mast cells resting, mast cells activated, and neutrophils. (c) The relationship between immune cells and signature combined with TMB expression on a string map showed that this combination significantly correlated with the expression of B cells memory, CD4 memory resting, M2 macrophages, mast cells resting, and neutrophils.

Recently, TMB has been found to be an important factor in cancer progression and immunity with increasing attention. Bi et al. showed that the gene mutation in ovarian cancer is very high [12]. In addition, study showed that ovarian cancer patients with higher TMB showed better overall survival [12]. According to TCGA gene mutations, we analyzed correlations among the top 20 mutational genes and the signature and found that no significant correlations existed (Table S9). Therefore, we could classify ovarian cancer patient statuses more specifically. Next, we performed a combined analysis of the signature with TMB expression in ovarian cancer patients. The OS of ovarian cancer patients with higher risk scores and lower TMB expression had the worst prognoses (Figure 5(a)). Moreover, the relationship of the signature or TMB expression with immune cells showed that the signature was significantly correlated with the expression of resting memory CD4 T cells, activated memory CD4 T cells, M0 macrophages, M2 macrophages, resting mast cells, activated mast cells, and neutrophils (Figure 5(b)). However, study showed that the expression of TMB also correlated with the expression of some kinds of immune cells in ovarian cancer patients, such as B cells naïve, B cells memory, T cells CD4 memory resting, T cells CD4 memory activated, T cells follicular helper, T cells regulatory, monocytes, macrophages M1, mast cells resting, and neutrophils [12]. Overall, these results demonstrated that the effects of the signature and TMB expression on immune cells were very different. Therefore, we combined the signature and TMB expression and found that memory B cells, resting memory CD4 T cells, M2 macrophages, resting mast cells, and neutrophils were closely correlated (Figure 5(c)). The expression of resting memory CD4 T cells, M2 macrophages, and neutrophils was positively correlated with the status of signatures combined with TMB expression in ovarian cancer patients.

## 3. Discussion

As a progressive disease, ovarian cancer needs reliable biomarkers to predict the prognosis and therapeutic targets. Currently, big data analyses are being used as a method to analyze cancer progression. The WGCNA algorithm is being used to analyze multivariate and highly complex data aside from other methods for the following reasons. First, the WGCNA algorithm focuses on coexpression rather than the expression of modules, and genes can be clustered into separate modules ^[^13^]^. This algorithm has been used to study the relationships between modules and clinical traits with high reliability from large and multidimensional datasets [14]. In our study, the ovarian cancer samples from TCGA dataset were used to investigate candidate prognostic-related genes via the WGCNA algorithm. Ovarian cancer patients are almost always diagnosed in the late stages of disease due to the anatomic location of the ovaries. The clinical stage, clinical grade, and the presence of lymphatic invasion significantly correlated with ovarian cancer prognoses. Therefore, according to the screening criteria, the turquoise and blue modules were viewed as the modules correlated with ovarian cancer prognoses.

Next, the Cox regression analyses were used to construct the signature that predicted the ovarian cancer prognoses. Cholesterol 25-hydroxylase (CH25H) correlated with immune responses and cells. Li et al. reported that CH25H and Liver X Receptor (LXR) stimulated by Krüppel-Like Factor 4 (KLF4) inhibited inflammation primarily through a decrease in inflammasome activity and promoted the repolarization of M1 to M2 macrophages [15]. HSPB7, a member of the heat-shock protein family, has been shown to function mainly in cardiac disease regulation; however, studies regarding its ability to regulate tumor progression are very limited. PPM2C, also known as pyruvate dehydrogenase phosphatase catalytic subunit 1 (PDP1), works primarily as an activator of Pyruvate Dehydrogenase E1 Subunit Alpha 1 (PDHA1) and Pyruvate Dehydrogenase Complex (PDC). Chen et al. reported that PDP1 was amplified and overexpressed in prostate tumors, promoting PDC control of lipid biosynthesis, further promoting prostate tumor progression [16]. However, no research has focused on studying LOC158830, also known as the CXorf65 gene. Our study used the Kaplan-Meier method and univariate Cox regression analysis to show that CH25H, HSPB7, and PPM2C correlated with ovarian cancer patients that had the worst prognoses. We also found that LOC158830 correlated ovarian cancer patients that had a better prognosis. We combined these four genes to construct a prognostic signature for ovarian cancer patients, which was validated by three independent cohorts. Thus, the results indicated that the signature could precisely predict ovarian cancer patient prognoses.

Immune cells have been shown to have a marked impact on cancer progression. To date, immunotherapies in ovarian cancer are still in the exploratory stages. However, an increasing number of in vitro and in vivo clinical immunotherapies have been investigated. Higuchi et al. reported that combined CTLA-4 antibody and PARP inhibitor therapy in BRCA1-deficient ovarian cancer patients significantly prolonged survival mediated by T cells [17]. In our study, we were first to find candidate genes and DEGs in high-risk vs. low-risk groups that significantly correlated with immune responses, which were confirmed with GO analyses. In addition, we found that our prognostic signature significantly correlated with the expression of various immune cells. IL10, IL6, IL6ST, and macrophage scavenger receptor 1 (MSR1; CD204) were positively correlated with ovarian cancer patients in the high-risk group using heatmap analyses of the signature with the immune-related genes. Among these molecules, IL10 works as a specific M2 macrophage marker. Miyasato et al. reported that macrophages with higher CD204 expression predicted worse prognoses in breast cancer patients [18]. Through peritoneal lavage component analyses of ovarian cancer, IL6 acted as an independent prognostic factor and correlated with the worst ovarian cancer prognoses[19]. The results of this research are consistent with that of our research.

Recent studies showed TMB did not only influence cancer prognoses, but higher expression of TMB also correlated with successful chemotherapy outcomes [20]. We also found that TMB expression correlated with ovarian cancer prognoses, although both the signature and TMB had a close relationship with prognoses and immune cell expression. However, since the relevance of TMB and our prognostic signature is unknown, we analyzed the combination of these two parameters with survival and immunity. The results showed that the combined analysis could more accurately demonstrate a patient's gene expression profile. The overall survival analysis showed that ovarian cancer patients with low TMB expression and high-risk signature scores had statistically worse prognoses compared with patients with high TMB expression and low-risk signature scores. In addition, resting memory CD4 T cells, M2 macrophages, and neutrophils were positively correlated with combined signature and TMB status. Other studies have shown that M2 macrophages and neutrophils promoted tumor progression in tumor microenvironments [21, 22]. Therefore, targeting these immune cells or inhibiting their expression might provide insights into ovarian cancer chemotherapies.

## 4. Methods

### 4.1. WGCNA Analysis

The RNA expression data and corresponding clinical information from TCGA database and GEO database were downloaded. The WGCNA package in R language was applied to evaluate the gene expression level and to test if they were good samples or good genes. Moreover, the flashClust package in the R language (http://www.r-project.org/) was used to conduct the clustering analysis of these samples. The power value in the module was sorted through the WGCNA algorithm. The independence and average degree of connectivity of various modules were identified with distinct power values via the gradient method. The best power value was determined when the independence degree was 0.9.

Pearson's correlation matrices were used to compare pair-wise genes. Using the power function a*mn* = ∣c*mn* | *β* (c*mn* = Pearson′s correlation between gene *m* and gene *n*; amn = adjacency between gene *m* and gene *n*), we constructed a weighted adjacency matrix. As a soft-thresholding parameter, *β* stresses the strong correlation between genes. After determining the appropriate power value, the genetic modules were constructed using the WGCNA algorithm with at least 50 genes in each module to regulate reliability, and the information of the corresponding genes in each module was extracted. Pearson's correlation matrices were used to compare pair-wise genes. Using the power function a*mn* = ∣c*mn* | *β* (c*mn* = Pearson′s correlation between gene *m* and gene *n*; a*mn* = adjacency between gene *m* and gene *n*), we constructed a weighted adjacency matrix. As a soft-thresholding parameter, *β* stresses the strong correlation between genes. After determining the appropriate power value, the genetic modules were constructed using the WGCNA algorithm with at least 50 genes in each module to regulate reliability, and the information of the corresponding genes in each module was extracted. The relationship between the module eigengenes (MEs) and clinical traits was identified in the module-trait relationships, which identified the relevant module for the clinical phenotype. Moreover, gene significance (GS), defined as the log10 transformation of the *p* value, was calculated for the specific clinical trait to evaluate the relationship between gene expression and the clinical traits using linear regression[23].

### 4.2. Bioinformatics Analysis

As an online program to analyze the functional annotation of the corresponding genes, DAVID (Database for Annotation, Visualization, and Integrated Discovery, http://david.abcc.ncifcrf.gov/) was used to conduct the Gene Ontology (GO) and Kyoto Encyclopedia of Genes and Genomes (KEGG) enrichment analyses. A heatmap of the signature with immune-related genes was analyzed using pheatmap in R package.

### 4.3. Statistical Analysis

The univariate Cox regression analysis was used to identify the candidate prognostic genes for ovarian carcinoma. The multivariate Cox regression analysis was performed to assess the signature with Akaike's information criterion (AIC) based on the candidate genes. Patients were divided into two groups according to the signature risk scores. The OS analysis was performed using the Kaplan-Meier method and compared with the log-rank test. The Pearson analysis was used to predict the correlation between the signature and the immune cells. A nomogram was constructed to predict patient prognoses for ovarian cancer patients based on the Cox regression model. One-way ANOVA was performed to evaluate the relationship between clinical parameters and relative expression levels. A ROC curve was prepared using the R package, survivalROC, to evaluate the signature's effect in ovarian cancer cases. The relationship between gene expression and the clinicopathologic characteristics was determined using the chi-square test or Fisher's exact test. The SPSS 18.0 (SPSS Inc., Chicago, IL, USA) and GraphPad Prism 7.0 (GraphPad Software, La Jolla, CA, USA) software were used for the statistical analyses. A *p* value < 0.05 was considered significant.

## 5. Conclusions

In summary, we investigated a novel prognostic signature that not only correlated with ovarian cancer prognoses but also correlated with the immune status of these cancer patients. Although our study still had the limitation of being retrospective, this prognostic signature could be used as a clinical tool to predict ovarian cancer patient prognoses and provide guidance for deciding on immunotherapy in ovarian cancer patients.

## Figures and Tables

**Table 1:** Top 15 genes significantly correlated with the overall survival of ovarian cancer patients.

Gene name	HR	*p* value
HSPB7	1.303	5.34 × 10^−5^
PPM2C	1.284	6.68 × 10^−5^
ZFHX4	1.216	8.69 × 10^−5^
ADH1B	1.223	9.77 × 10^−5^
CH25H	1.180	0.000164
GFPT2	1.257	0.000232
OGN	1.124	0.000257
SUSD5	1.218	0.000262
CCDC80	1.202	0.000311
ZNF521	1.129	0.000314
PHLDB2	1.226	0.000361
PTGER3	1.242	0.000538
C1QTNF7	1.209	0.000571
LOC158830	0.799	0.000641
PTGIS	1.137	0.000648

**Table 2:** The univariate and multivariate Cox regression analysis of the signature with clinical characteristics predictive of overall survival in ovarian cancer in TCGA cohort.

Variable	Overall survival			
	Univariate		Multivariate	
	HR	*p* value	HR	*p* value
Signature (low-risk vs. high-risk)	0.438	<0.001	0.575	<0.001
Lymphatic invasion (invasion vs. noninvasion)	1.422	0.114		
Grade (G3-G4 vs. G1-G2)	1.181	0.054		
Clinical stage (stage III-IV vs. stage I-II)	1.660	0.003	1.602	0.026
Tumor residual disease (visible macroscopic vs. no macroscopic)	2.295	<0.001	1.775	0.006
Venous invasion (noninvasion vs. invasion)	0.971	0.910		
Age (old age vs. young age)	1.342	0.015	1.286	0.050

**Table 3:** Relationship of clinical characteristics of ovarian cancer patients and signature in TCGA cohort.

Characteristics	High-risk (*n* = 266)	Low-risk (*n* = 266)	*p* value
Age			0.528
Mean (years)	59.27 ± 0.72	59.91 ± 0.71	
Stage			0.033
I	4	11	
II	9	16	
III	202	208	
IV	48	30	
NA	3	1	
Grade			0.123
G1	5	1	
G2	40	27	
G3	214	232	
G4	0	1	
NA	7	5	
Residual tumor size			<0.001
No macroscopic	36	70	
1-10 mm	134	100	
11-20 mm	20	14	
>20 mm	55	45	
NA	21	37	
Lymphatic invasion			0.067
Yes	75	57	
No	31	47	
NA	160	163	
Venous invasion			0.003
Yes	52	34	
No	22	45	
NA	192	187	
KPS			0.058
40	0	2	
60	7	8	
80	13	27	
100	2	5	
NA	244	224	
Therapy outcome			0.006
Complete remission/response	133	165	
Partial remission/response	40	20	
Progressive disease	24	12	
Sable disease	14	16	
NA	55	53	
Neoplasm cancer status			0.002
With tumor	184	150	
Tumor free	50	85	
NA	32	31	
New neoplasm event type			0.081
Locoregional disease	5	1	
Metastatic	1	0	
Progression of disease	12	15	
Recurrence	143	121	
NA	105	129	

## Data Availability

The data that support the findings of this study are available from the corresponding author upon reasonable request.

## Supplementary Materials

Supplementary Materials Figure S1: identification of clustering in ovarian cancer samples. (a) Sample clustering of TCGA samples to detect outliers. (b) A sample dendrogram and trait indicator. The clustering was based on the gene expression data. The color intensity was proportional to higher clinical stages and grades. For lymphatic invasion, white indicates no lymphatic invasion, and red indicates lymphatic invasion. Figure S2: the determination of a soft-thresholding power in weighted gene coexpression network analysis (WGCNA). (a) Screening for the appropriate soft-thresholding power in WGCNA. An analysis of the scale-free fit index for various soft-thresholding powers (*β*). An analysis of the mean connectivity for various soft-thresholding powers. (b) A histogram of the connectivity distribution when *β* = 3. Scale-free topology when *β* = 3. Figure S3: Gene Ontology enrichment analysis and KEGG pathway enrichment analysis of genes in the turquoise and blue modules. The size of the balls represents the *p* value of each term, the smaller the *p* value, the bigger the size. *Y*-axis represents the number of related genes. (a) Biological process enrichment. (b) Molecular function enrichment. (c) Cellular component enrichment. (d) KEGG pathway enrichment. Figure S4: the Kaplan-Meier curve for overall survival of candidate genes in ovarian cancer. (a) HSPB7, (b) PPM2C, (c) ZFHX4, (d) ADH1B, (e) CH25H, (f) GFPT2, (g) OGN, (h) SUSD5, (i) CCDC80. (j) ZNF521, (k) PHLDB2, (l) PTGER3, (m) C1QTNF7, (n) LOC158830, and (o) PTGIS. Figure S5: the overall survival of the signature in the cohorts stratified by grades, ages, and residual tumor sizes. (a) Low grade (G1-G2) of ovarian cancer patients. (b) High grade (G3-G4) of ovarian cancer patients. (c) Young age of ovarian cancer patients. (d) Old age of ovarian cancer patients. (e) No macroscopic of residual tumor size in ovarian cancer patients. (f) 1-10 mm of residual tumor size in ovarian cancer patients. (g) 11-20 mm of residual tumor size in ovarian cancer patients. (h) >20 mm residual tumor size in ovarian cancer patients. Figure S6: Gene Ontology (GO) enrichment analysis and Kyoto Encyclopedia of Genes and Genomes (KEGG) pathway enrichment analyses of differently expressed genes in the signature. The size of the balls represents the number of related genes. The color of the balls represents the *p* value of each term; the red color indicates the smallest *p* value. (a) Biological process enrichment. (b) Cellular component enrichment. (c) Molecular function enrichment. (d) KEGG pathway enrichment. Table S1: the details of separate modules. Table S2: the main biological process in the genes from blue and turquoise modules. Table S3: the main cellular component in the genes from blue and turquoise modules. Table S4: the main molecular function in the genes from blue and turquoise modules. Table S5: the main KEGG pathway in the genes from blue and turquoise modules. Table S6: the DEGs between high-risk group and low-risk group. Table S7: the univariate and multivariate Cox regression analysis of signature and clinical characteristics predictive of overall survival in validation cohorts. Table S8: clinical characteristics of ovarian cancer patients by signature in validation cohorts. Table S9: gene mutation status in ovarian cancer according to the signature divided by risk score.
