# Supplementary material for: Development and Validation of an Immune-Related Prognostic Signature for Ovarian Cancer Based on Weighted Gene Coexpression Network Analysis
Source: Biomed Res Int. 2020 Dec 10;2020:7594098. doi: 10.1155/2020/7594098 (PMC7749778; doi:10.1155/2020/7594098)
Supplement: Supplementary Materials — Figure S1: identification of clustering in ovarian cancer samples. (a) Sample clustering of TCGA samples to detect outliers. (b) A sample dendrogram and trait indicator. The clustering was based on the gene expression data. The color intensity was proportional to higher clinical stages and grades. For lymphatic invasion, white indicates no lymphatic invasion, and red indicates lymphatic invasion. Figure S2: the determination of a soft-thresholding power in weighted gene coexpression network analysis (WGCNA). (a) Screening for the appropriate soft-thresholding power in WGCNA. An analysis of the scale-free fit index for various soft-thresholding powers (β). An analysis of the mean connectivity for various soft-thresholding powers. (b) A histogram of the connectivity distribution when β = 3. Scale-free topology when β = 3. Figure S3: Gene Ontology enrichment analysis and KEGG pathway enrichment analysis of genes in the turquoise and blue modules. The size of the balls represents the p value of each term, the smaller the p value, the bigger the size. Y-axis represents the number of related genes. (a) Biological process enrichment. (b) Molecular function enrichment. (c) Cellular component enrichment. (d) KEGG pathway enrichment. Figure S4: the Kaplan-Meier curve for overall survival of candidate genes in ovarian cancer. (a) HSPB7, (b) PPM2C, (c) ZFHX4, (d) ADH1B, (e) CH25H, (f) GFPT2, (g) OGN, (h) SUSD5, (i) CCDC80. (j) ZNF521, (k) PHLDB2, (l) PTGER3, (m) C1QTNF7, (n) LOC158830, and (o) PTGIS. Figure S5: the overall survival of the signature in the cohorts stratified by grades, ages, and residual tumor sizes. (a) Low grade (G1-G2) of ovarian cancer patients. (b) High grade (G3-G4) of ovarian cancer patients. (c) Young age of ovarian cancer patients. (d) Old age of ovarian cancer patients. (e) No macroscopic of residual tumor size in ovarian cancer patients. (f) 1-10 mm of residual tumor size in ovarian cancer patients. (g) 11-20 mm of residual tumor size in ovarian cance [file 7594098.f1.zip › 7594098.f2.docx]

**Table S1 The details of separate modules**

| Sequence of the module | Color of the module | Gene number of the module |
| --- | --- | --- |
| Module 1 | Grey module | 1898 |
| Module 2 | Turquoise module | 1020 |
| Module 3 | Blue module | 628 |
| Module 4 | Brown module | 246 |
| Module 5 | Yellow module | 195 |
| Module 6 | Green module | 185 |
| Module 7 | Red module | 112 |
| Module 8 | Black module | 89 |
| Module 9 | Pink module | 81 |

**Table S2 The main biological process in the genes from blue and turquoise modules**

| **Terms** | **Count** | ***p*-value** |
| --- | --- | --- |
| Immune response | 126 | 6.50×10^-40^ |
| Inflammatory response | 116 | 9.30×10^-38^ |
| Extracellular matrix organization | 73 | 8.11×10^-30^ |
| Cell adhesion | 110 | 9.68×10^-26^ |
| Chemokine-mediated signaling pathway | 33 | 4.55×10^-17^ |
| Skeletal system development | 46 | 5.65×10^-17^ |
| Interferon-gamma-mediated signaling pathway | 32 | 4.24×10^-16^ |
| Positive regulation of inflammatory response | 32 | 1.21×10^-15^ |
| Signal transduction | 172 | 1.87×10^-15^ |
| Defense response to virus | 48 | 6.97×10^-15^ |
| Cellular response to lipopolysaccharide | 39 | 7.43×10^-15^ |
| Response to virus | 37 | 9.78×10^-14^ |
| Response to lipopolysaccharide | 46 | 1.16×10^-13^ |
| Cell-cell signaling | 59 | 2.24×10^-13^ |
| Angiogenesis | 54 | 4.53×10^-13^ |
| Chemotaxis | 38 | 6.32×10^-13^ |
| Cellular response to tumor necrosis factor | 35 | 2.99×10^-12^ |
| Positive regulation of ERK1 and ERK2 cascade | 45 | 5.79×10^-12^ |
| Leukocyte migration | 36 | 1.56×10^-11^ |
| Innate immune response | 78 | 1.82×10^-11^ |
| Regulation of immune response | 44 | 4.14×10^-11^ |
| Positive regulation of gene expression | 54 | 3.07×10^-10^ |
| Cell surface receptor signaling pathway | 53 | 4.67×10^-09^ |
| Positive regulation of angiogenesis | 31 | 5.27×10^-09^ |
| Positive regulation of cell migration | 40 | 1.76×10^-08^ |
| Adaptive immune response | 34 | 6.10×10^-08^ |

**Table S3 The main cellular component in the genes from blue and turquoise modules**

| **Terms** | **Count** | ***p*-value** |
| --- | --- | --- |
| Extracellular space | 286 | 6.50×10^-59^ |
| Extracellular region | 290 | 2.34×10^-44^ |
| Proteinaceous extracellular matrix | 89 | 1.08×10^-32^ |
| Integral component of plasma membrane | 231 | 2.97×10^-28^ |
| Cell surface | 119 | 3.74×10^-25^ |
| Extracellular matrix | 83 | 6.47×10^-25^ |
| External side of plasma membrane | 67 | 3.14×10^-23^ |
| Plasma membrane | 465 | 1.92×10^-19^ |
| Collagen trimer | 33 | 1.76×10^-13^ |
| Extracellular exosome | 319 | 9.86×10^-13^ |
| Endoplasmic reticulum lumen | 41 | 9.80×10^-09^ |
| Integral component of membrane | 500 | 1.34×10^-08^ |
| Basement membrane | 20 | 8.40×10^-06^ |
| Membrane raft | 36 | 1.15×10^-05^ |
| Golgi lumen | 20 | 1.48×10^-04^ |
| Focal adhesion | 51 | 4.35×10^-04^ |
| Golgi apparatus | 95 | 7.33×10^-04^ |
| Blood microparticle | 25 | 7.80×10^-04^ |
| Receptor complex | 22 | 8.83×10^-04^ |
| Lamellipodium | 24 | 3.47×10^-03^ |
| Lysosome | 30 | 6.08×10^-03^ |
| Golgi membrane | 64 | 8.22×10^-03^ |

**Table S4 The main molecular function in the genes from blue and turquoise modules**

| **Terms** | **Count** | ***p*-value** |
| --- | --- | --- |
| Heparin binding | 53 | 1.29×10^-19^ |
| Chemokine activity | 26 | 1.95×10^-15^ |
| Extracellular matrix structural constituent | 30 | 3.00×10^-15^ |
| Receptor activity | 48 | 1.29×10^-10^ |
| Calcium ion binding | 102 | 5.11×10^-09^ |
| Carbohydrate binding | 42 | 5.75×10^-09^ |
| Collagen binding | 21 | 1.42×10^-08^ |
| Cytokine activity | 38 | 2.74×10^-08^ |
| Growth factor activity | 35 | 1.04×10^-07^ |
| Integrin binding | 25 | 1.55×10^-06^ |
| Receptor binding | 55 | 1.71×10^-06^ |
| Transmembrane signaling receptor activity | 38 | 4.36×10^-06^ |
| Serine-type endopeptidase activity | 42 | 8.76×10^-06^ |
| Protease binding | 21 | 1.00×10^-04^ |
| Identical protein binding | 83 | 1.33×10^-03^ |
| Protein homodimerization activity | 81 | 1.47×10^-03^ |
| Transcriptional activator activity, RNA polymerase II core promoter proximal region sequence-specific binding | 31 | 6.17×10^-03^ |

**Table S5 The main KEGG pathway in the genes from blue and turquoise modules**

| **Terms** | **Count** | ***p*-value** |
| --- | --- | --- |
| Cytokine-cytokine receptor interaction | 73 | 2.07×10^-19^ |
| Cell adhesion molecules (CAMs) | 50 | 1.78×10^-16^ |
| Staphylococcus aureus infection | 29 | 3.48×10^-15^ |
| Rheumatoid arthritis | 37 | 5.30×10^-15^ |
| Inflammatory bowel disease (IBD) | 29 | 8.07×10^-13^ |
| Leishmaniasis | 30 | 2.61×10^-12^ |
| ECM-receptor interaction | 32 | 3.17×10^-11^ |
| Antigen processing and presentation | 28 | 6.54×10^-10^ |
| Phagosome | 41 | 1.17×10^-09^ |
| Chemokine signaling pathway | 46 | 3.16×10^-09^ |
| Hematopoietic cell lineage | 29 | 4.16×10^-09^ |
| Influenza A | 43 | 1.16×10^-08^ |
| Protein digestion and absorption | 27 | 1.07×10^-07^ |
| Tuberculosis | 40 | 5.17×10^-07^ |
| HTLV-I infection | 51 | 5.30×10^-07^ |
| Focal adhesion | 44 | 6.55×10^-07^ |
| PI3K-Akt signaling pathway | 63 | 6.68×10^-07^ |
| Herpes simplex infection | 40 | 1.27×10^-06^ |
| Amoebiasis | 28 | 1.63×10^-06^ |
| TNF signaling pathway | 28 | 1.98×10^-06^ |
| Pathways in cancer | 67 | 3.45×10^-06^ |
| Toxoplasmosis | 27 | 1.11×10^-05^ |
| Toll-like receptor signaling pathway | 26 | 1.71×10^-05^ |
| Proteoglycans in cancer | 39 | 2.92×10^-05^ |
| Measles | 29 | 5.08×10^-05^ |

**Table S6 The DEGs between high-risk group and low-risk group**

|  | LogFC | P-value |
| --- | --- | --- |
| CAPZA2 | -0.94062 | 1.72E-31 |
| GPR34 | -1.63066 | 5.71E-27 |
| SLC35A5 | -0.68952 | 5.71E-27 |
| LIPA | -0.74031 | 1.52E-26 |
| MTMR6 | -0.62797 | 3.16E-26 |
| hCG_1757335 | -0.78754 | 1.61E-24 |
| LYPLA1 | -0.8076 | 2.25E-24 |
| FLJ11171 | -0.625 | 2.89E-24 |
| IMPA1 | -0.67912 | 1.33E-23 |
| LMNB1 | 1.105942 | 9.82E-23 |
| CDC42SE2 | -0.63259 | 9.82E-23 |
| NCF4 | -0.81542 | 1.6E-22 |
| C14orf129 | -0.71742 | 1.86E-22 |
| RBM7 | -0.87899 | 2.9E-22 |
| TMED2 | -0.69474 | 2.9E-22 |
| CRBN | -0.60168 | 7.3E-22 |
| ALOX5AP | -1.26732 | 8.16E-22 |
| F13A1 | -1.12482 | 8.16E-22 |
| MORF4 | -0.62416 | 1.51E-21 |
| ITGA4 | -0.87906 | 1.74E-21 |
| PHACTR4 | 0.586564 | 3.09E-21 |
| TLR4 | -0.89446 | 3.37E-21 |
| CLEC4A | -0.96242 | 3.83E-21 |
| VPS26A | -0.62314 | 3.83E-21 |
| MS4A4A | -1.11269 | 4.58E-21 |
| SNAP23 | -0.6984 | 1.37E-20 |
| FOLR2 | -0.6382 | 7.93E-20 |
| DNAJB9 | -0.62272 | 8.74E-20 |
| C3AR1 | -0.85133 | 3.13E-19 |
| SCOC | -0.66758 | 3.9E-19 |
| UBE2Q2 | -0.6711 | 5.1E-19 |
| NPL | -0.68154 | 2.61E-18 |
| SMPDL3A | -0.77296 | 2.72E-18 |
| MSR1 | -0.91626 | 1.27E-17 |
| MS4A6A | -0.87344 | 1.51E-17 |
| SPP1 | -1.13506 | 2.22E-17 |
| EVI2A | -1.0087 | 3.44E-17 |
| RCHY1 | -0.64881 | 3.61E-17 |
| P2RY5 | -0.87273 | 4.67E-17 |
| MS4A7 | -0.82922 | 7.58E-17 |
| GOLT1B | -0.60488 | 7.58E-17 |
| IDI1 | -0.65291 | 9.01E-17 |
| ZFAND1 | -0.6219 | 9.74E-17 |
| FCGR2B | -1.07318 | 1.1E-16 |
| IGSF6 | -1.00585 | 1.1E-16 |
| INCENP | 0.585516 | 1.88E-16 |
| COMMD10 | -0.59934 | 3.03E-16 |
| CD300LF | -0.60847 | 5.42E-16 |
| SLCO2B1 | -0.86473 | 7.33E-16 |
| TMEM182 | -0.61826 | 7.68E-16 |
| SPRED1 | -0.59371 | 1.1E-15 |
| PGDS | -0.89355 | 1.57E-15 |
| MINPP1 | -0.65802 | 1.78E-15 |
| HCK | -0.74712 | 3.9E-15 |
| TFEC | -0.88835 | 5.08E-15 |
| ARHGAP15 | -0.70043 | 5.16E-15 |
| SLC35B3 | -0.63579 | 6.01E-15 |
| ADORA3 | -0.72716 | 6.57E-15 |
| SRGN | -0.72032 | 1.15E-14 |
| NXT2 | -0.69495 | 1.16E-14 |
| LOC402176 | -0.59947 | 2.34E-14 |
| GIMAP6 | -0.61505 | 2.96E-14 |
| CD53 | -0.87045 | 3.05E-14 |
| TMEM64 | -0.63527 | 3.43E-14 |
| CXorf21 | -0.77073 | 4.06E-14 |
| VNN2 | -0.93302 | 4.13E-14 |
| ATP8B4 | -0.64119 | 5.22E-14 |
| CCNC | -0.59754 | 6.67E-14 |
| TLR7 | -0.671 | 6.76E-14 |
| FPRL2 | -0.81729 | 8.44E-14 |
| RGS18 | -0.8187 | 9.82E-14 |
| GIMAP2 | -0.81031 | 1.27E-13 |
| CD28 | -0.65229 | 1.55E-13 |
| C4orf18 | -0.78554 | 1.74E-13 |
| MAF | -0.70166 | 1.9E-13 |
| GPR65 | -0.96317 | 1.91E-13 |
| CTSO | -0.67059 | 2.07E-13 |
| ZNF420 | -0.62003 | 4.18E-13 |
| FPRL1 | -0.6632 | 4.58E-13 |
| AOAH | -0.70686 | 6.14E-13 |
| PTPRC | -0.79125 | 6.63E-13 |
| SAMSN1 | -0.75578 | 7.99E-13 |
| CPA3 | -0.99575 | 8.34E-13 |
| CCL13 | -1.0943 | 8.49E-13 |
| EDIL3 | -0.92302 | 2.22E-12 |
| CD163 | -0.83962 | 2.47E-12 |
| HNMT | -0.63165 | 3.48E-12 |
| LAIR1 | -0.65075 | 4.87E-12 |
| GATM | -0.78796 | 4.9E-12 |
| PLEK | -0.68777 | 5.06E-12 |
| TLR8 | -0.79431 | 6.88E-12 |
| CD33 | -0.62254 | 8.36E-12 |
| LYZ | -1.01679 | 1.23E-11 |
| FCGR2A | -0.69219 | 1.58E-11 |
| FCGR3A | -0.7033 | 1.62E-11 |
| RNASE6 | -0.67359 | 1.63E-11 |
| TREM1 | -0.61801 | 2.26E-11 |
| ECM2 | -0.8513 | 2.43E-11 |
| FGL2 | -0.80575 | 3.46E-11 |
| NLRC4 | -0.70094 | 3.78E-11 |
| MNDA | -0.83398 | 4.29E-11 |
| CD48 | -0.88102 | 4.35E-11 |
| PKD2L1 | -0.70474 | 5.39E-11 |
| CCR2 | -0.72061 | 6.52E-11 |
| LAPTM5 | -0.63106 | 1.39E-10 |
| CTSS | -0.65363 | 1.89E-10 |
| CLC | -0.62267 | 2.31E-10 |
| FAS | -0.59116 | 2.31E-10 |
| KCNK13 | -0.63239 | 2.38E-10 |
| HTR2B | -0.75012 | 2.38E-10 |
| TLR1 | -0.63703 | 3.03E-10 |
| AIF1 | -0.69866 | 3.6E-10 |
| TAGAP | -0.63158 | 4.24E-10 |
| GGTA1 | -0.78095 | 4.36E-10 |
| TLR2 | -0.65599 | 6.33E-10 |
| EBI2 | -0.81936 | 6.64E-10 |
| CCL18 | -0.80232 | 7.15E-10 |
| DCN | -0.95453 | 7.55E-10 |
| CRTAM | -0.62591 | 9.77E-10 |
| CYP39A1 | -0.60272 | 1.4E-09 |
| C5orf29 | -0.7021 | 1.77E-09 |
| APOBEC3G | -0.58612 | 2.49E-09 |
| GPR18 | -0.65579 | 2.51E-09 |
| ELTD1 | -0.59208 | 2.6E-09 |
| TREM2 | -0.60362 | 2.67E-09 |
| CLEC4E | -0.75481 | 2.92E-09 |
| HS3ST1 | -0.60361 | 3.24E-09 |
| EMB | -0.59072 | 5.63E-09 |
| KIAA1913 | -0.64833 | 5.85E-09 |
| ADAMDEC1 | -1.1401 | 5.91E-09 |
| CYBB | -0.70275 | 9.57E-09 |
| MATN3 | -0.63013 | 1.23E-08 |
| EVI2B | -0.58779 | 2.6E-08 |
| PKIB | -0.67079 | 5.01E-08 |
| OMD | -0.67335 | 8.14E-08 |
| TRIM22 | -0.59131 | 1.25E-07 |
| CLEC2B | -0.65561 | 1.55E-07 |
| ANKRD22 | -0.74792 | 1.61E-07 |
| MARCO | -0.64265 | 1.85E-07 |
| IL1B | -0.60572 | 3.24E-07 |
| KCND2 | -0.59469 | 3.97E-07 |
| C15orf48 | -0.69733 | 4.72E-07 |
| CTSK | -0.76243 | 5.56E-07 |
| CCL8 | -0.62502 | 8.46E-07 |
| GPNMB | -0.63172 | 1.1E-06 |
| KLRB1 | -0.71371 | 1.29E-06 |
| FCER1A | -0.72992 | 1.39E-06 |
| CFHR3 | -0.67536 | 1.46E-06 |
| GJA1 | -0.60853 | 1.73E-06 |
| MMP12 | -0.99269 | 1.9E-06 |
| IL7 | -0.5996 | 2.03E-06 |
| ASPN | -0.90385 | 3.52E-06 |
| LUM | -0.74915 | 8.11E-06 |
| OGN | -0.81304 | 1.16E-05 |
| TDO2 | -0.65245 | 1.59E-05 |
| C6orf15 | -0.6489 | 1.62E-05 |
| IGKC | -0.82199 | 1.68E-05 |
| CDH11 | -0.62995 | 3.26E-05 |
| CTHRC1 | -0.64872 | 4.74E-05 |
| SLAMF7 | -0.58748 | 5.8E-05 |
| IGFL2 | -0.78777 | 6.28E-05 |
| POSTN | -1.05186 | 0.000103 |
| SFRP4 | -0.71635 | 0.000125 |
| DDIT4L | -0.59072 | 0.000129 |
| IGKV1-5 | -0.76277 | 0.00017 |
| RARRES1 | -0.58577 | 0.000623 |
| SCRG1 | -0.59683 | 0.000699 |
| FAP | -0.70404 | 0.000972 |
| CXCL9 | -0.61208 | 0.001242 |
| MAGEC2 | 0.736645 | 0.001963 |

**Table S7 Univariate and multivariate Cox regression analysis of signature and clinical characteristics predictive of overall survival in validation cohorts**

| Variable | | Overall survival | | | |
| --- | --- | --- | --- | --- | --- |
|  |  | Univariate | | Multivariate | |
|  |  | HR | *p*-value | HR | *p*-value |
| *GSE26193* | Signature  (Low-risk vs High-risk) | 0.461 | 0.030 | 0.491 | 0.046 |
|  | Grade  (G3-G4 vs G1-G2) | 1.063 | 0.801 |  |  |
|  | Stage  (Stage III-IV vs Stage I-II) | 4.197 | <0.001 | 4.107 | <0.001 |
| *GSE63885* | Signature  (Low-risk vs High-risk) | 0.398 | 0.035 | 0.459 | 0.078 |
|  | Grade  (G3-G4 vs G1-G2) | 2.263 | 0.046 | 1.944 | 0.109 |
|  | Stage  (Stage III-IV vs Stage I-II) | 1.606 | 0.640 |  |  |
|  | BRCA1 mutation  (Yes vs No) | 0.803 | 0.440 |  |  |
|  | TP53 mutation  (Yes vs No) | 1.684 | 0.201 |  |  |
|  | TP53 accumulation  (Yes vs No) | 1.129 | 0.632 |  |  |

**Table S8 Clinical characteristics of ovarian cancer patients by signature in validation cohorts**

| Dataset | Characteristics | High-risk | Low-risk | *p*-value |
| --- | --- | --- | --- | --- |
| *GSE26193* | Stage  I  II  III  IV | 9  5  28  12 | 11  6  31  5 | 0.345 |
|  | Grade  G1  G2  G3 | 3  15  36 | 4  18  31 | 0.677 |
| *GSE63885* | BRCA1 mutation  Yes  No | 12  23 | 8  27 | 0.290 |
|  | TP53 mutation  Yes  No | 30  5 | 32  3 | 0.452 |
|  | TP53 accumulation  Yes  No | 22  13 | 19  16 | 0.467 |
|  | Grade  G2  G3  G4 | 4  19  12 | 4  25  6 | 0.244 |
|  | Stage  II  III  IV | 1  27  7 | 0  32  3 | 0.220 |
|  | Residual tumor size  < 1cm  1-5cm  > 5cm | 7  19  9 | 7  17  11 | 0.856 |
|  | Chemotherapy  Complete response  Partial response  Progression  Stable disease | 23  7  3  2 | 22  8  4  1 | 0.904 |
|  | Platinum sensitivity  Highly sensitive  Moderately sensitive  Resistant | 4  13  18 | 6  13  16 | 0.772 |

**Table S9 Gene mutation status in ovarian cancer according to the signature divided by risk score**

|  | | High-risk (n=191) | Low-risk (n=191) | *p*-value |
| --- | --- | --- | --- | --- |
| APOB | Mutation | 12  179 | 16  175 | 0.432 |
|  | No mutation |  |  |  |
| BRCA1 | Mutation | 8  183 | 10  181 | 0.629 |
|  | No mutation |  |  |  |
| CDK12 | Mutation | 7  184 | 11  180 | 0.334 |
|  | No mutation |  |  |  |
| COL6A3 | Mutation | 8  183 | 12  179 | 0.358 |
|  | No mutation |  |  |  |
| CSMD3 | Mutation | 14  177 | 19  172 | 0.363 |
|  | No mutation |  |  |  |
| DNAH3 | Mutation | 10  181 | 12  179 | 0.660 |
|  | No mutation |  |  |  |
| FAT3 | Mutation | 14  177 | 16  175 | 0.704 |
|  | No mutation |  |  |  |
| FLG | Mutation | 14  177 | 12  179 | 0.685 |
|  | No mutation |  |  |  |
| HMCN1 | Mutation | 13  178 | 19  172 | 0.268 |
|  | No mutation |  |  |  |
| LRP1B | Mutation | 11  180 | 9  182 | 0.646 |
|  | No mutation |  |  |  |
| MACF1 | Mutation | 10  181 | 16  175 | 0.223 |
|  | No mutation |  |  |  |
| MUC16 | Mutation | 20  171 | 19  172 | 0.866 |
|  | No mutation |  |  |  |
| MUC17 | Mutation | 15  176 | 17  174 | 0.712 |
|  | No mutation |  |  |  |
| NF1 | Mutation | 16  175 | 13  178 | 0.562 |
|  | No mutation |  |  |  |
| RB1 | Mutation | 5  186 | 12  179 | 0.082 |
|  | No mutation |  |  |  |
| RYR2 | Mutation | 13  178 | 15  176 | 0.695 |
|  | No mutation |  |  |  |
| TOP2A | Mutation | 14  177 | 11  180 | 0.535 |
|  | No mutation |  |  |  |
| TP53 | Mutation | 183  8 | 183  8 | 1 |
|  | No mutation |  |  |  |
| TTN | Mutation | 78  113 | 77  114 | 0.917 |
|  | No mutation |  |  |  |
| USH2A | Mutation | 16  175 | 13  178 | 0.562 |
|  | No mutation |  |  |  |
